# Aberrant Origin of the Right Inferior Thyroid Artery From the Common Carotid Artery Associated With Contralateral Absence

**DOI:** 10.7759/cureus.107984

**Published:** 2026-04-29

**Authors:** Maria Piagkou, George Triantafyllou

**Affiliations:** 1 Department of Anatomy, School of Medicine, Faculty of Health Sciences, National and Kapodistrian University of Athens, Athens, GRC

**Keywords:** anatomical variation, cadaver dissection, common carotid artery, inferior thyroid artery, thyroid gland

## Abstract

Variations of the inferior thyroid artery (ITA) are clinically significant due to their close anatomical relationship with the recurrent laryngeal nerve (RLN) and their relevance in anterior neck surgery. During routine cadaveric dissection of a 67-year-old male donor, an unusual thyroid arterial configuration was identified. The right ITA (RITA) arose aberrantly from the right common carotid artery (CCA), while the left ITA was absent. An accessory right thyroid lobe was also present. The aberrant artery supplied the right lobe, the accessory lobe, and, through an extensive isthmic and interlobar arterial network, the left lobe, compensating for the absent contralateral ITA. The superior thyroid arteries exhibited a typical bilateral origin. This case highlights an extremely uncommon combination of thyroid arterial variations with important surgical implications. Preoperative computed tomography angiography may facilitate safe surgical planning.

## Introduction

Typically, the thyroid gland receives its arterial supply from the superior and inferior thyroid arteries (STA and ITA). In most individuals, the STA arises from the external carotid artery (ECA), whereas the ITA originates from the thyrocervical trunk of the subclavian artery (SCA) [1-4]. Large-scale radioanatomical studies and meta-analyses have extensively investigated the origin patterns of the STA [1,2] and ITA [3,4], highlighting both their common configurations and anatomical variability.

Bergman’s Comprehensive Encyclopedia of Human Anatomic Variations describes several variants of the ITA, including complete absence and atypical origins [5]. The reported incidence of ITA absence ranges from 1% to 6% across studies, while its origin from the thyrocervical trunk has been documented in approximately 85-95% of cases [5]. Rarely, the ITA may arise from the common carotid artery (CCA), aortic arch, brachiocephalic trunk, internal thoracic artery, or vertebral artery [5]. Herein, we report the coexistence of an aberrant origin of the right ITA from the CCA with the absence of the contralateral ITA, identified incidentally during routine cadaveric dissection.

## Case presentation

Routine dissection was performed on a 67-year-old male cadaver for educational and research purposes. During the procedure, the thyroid gland, along with the major cervical arteries, was removed en bloc. The specimen was obtained through the “Body Donation Program” of the responsible authorities (affiliation 1), in accordance with institutional and ethical guidelines [6]. An unusual vascular variation was identified during detailed examination of the specimen.

The thyroid gland consisted of the right and left lobes and the isthmus. An accessory right thyroid lobe was present, located inferomedially to the right lobe and connected to the main gland by thyroid parenchyma. The STAs arose bilaterally from the ECA and followed a typical descending course to supply the superior poles of both lobes.

**Figure 1:**
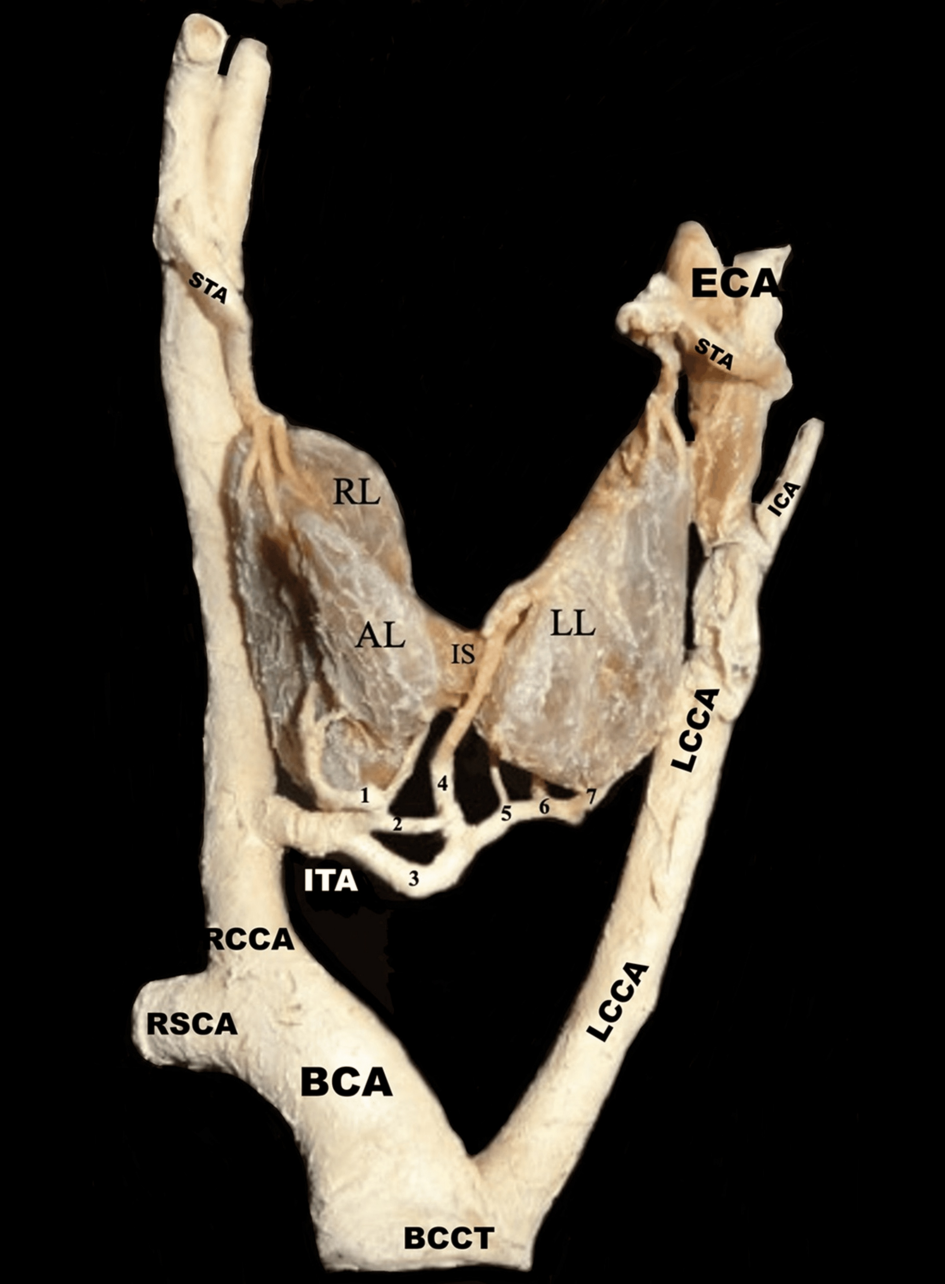
Anterior view of the en-bloc dissection showing an aberrant right ITA arising from the RCCA with the absence of the left ITA ITA: inferior thyroid artery; BCA: brachiocephalic artery; RSCA: right subclavian artery; LCCA: left common carotid artery; RCCA: right common carotid artery; RL: right thyroid lobe; AL: accessory right thyroid lobe; IS: isthmus; LL: left thyroid lobe; STAs: superior thyroid arteries; ICA: internal carotid artery; ECA: external carotid artery; BCCT: brachiocephalico-carotid trunk The aberrant ITA supplies the RL, an AL, and the IS, forming an extensive interlobar arterial anastomotic network contributing to the vascularization of the LL. Branches labeled 1-7 represent isthmic and interlobar anastomotic vessels, while branches a and b correspond to glandular branches of the ITA supplying the RL and AL. STAs show a typical bilateral origin

Inferiorly, a variant ITA was identified arising directly from the right CCA. After its origin, the right ITA coursed medially and superiorly toward the thyroid gland, giving rise to multiple branches. Glandular branches supplied the right thyroid lobe and the accessory right lobe. The artery is further divided into several isthmic and interlobar branches that form an extensive arterial anastomotic network across the isthmus, providing vascular communication between the right and left thyroid lobes. These branches contributed significantly to the arterial supply of the left thyroid lobe, compensating for the absence of the left ITA (Figure 1).

## Discussion

From an embryological perspective, this variation may be explained by the complex remodeling of the aortic arch system [7]. The vascular supply of the thyroid gland is established during its descent from the foramen caecum [7]. The STA derives from the ECA (a derivative of the third aortic arch) [2], while the ITA originates from the subclavian artery (derived in part from the seventh intersegmental artery) [4]. In our case, the origin of the ITA from the CCA likely represents the persistence of a primitive branch from the third aortic arch that failed to regress, combined with failure of the typical thyrocervical trunk-derived supply to develop. The coexistence of a contralateral ITA absence further suggests that the right-sided aberrant vessel underwent compensatory enlargement to sustain the entire gland’s supply.

The aberrant origin of the ITA from the CCA, observed in the current case, is considered rare according to Bergman’s Comprehensive Encyclopedia of Human Anatomic Variations, while the ITA absence was also uncommon [5].

These findings are corroborated by the systematic review conducted by Toni et al. [8], which analyzed 33 anatomical studies encompassing over 2,000 specimens. In this publication, they reported only six instances of the ITA originating from the CCA out of the 33 studies analyzed [8]. The rarity of this finding is further supported by the recent systematic review by Bruna-Mejias et al. [4], which concluded that the ITA arising from the CCA is rare.

However, it is important to note that these sporadic cases have not been described in association with the absence of the contralateral ITA, unlike the current case. In such cases, the arterial flow to the gland can be replaced either via the STA [7], the contralateral ITA, or a variant thyroidea ima artery (TIA) [9].

It is essential to differentiate the current finding from a TIA, also recognized as the artery of Neubauer [10,11]. Although both entities are arterial variations that supply the inferior thyroid gland, they are anatomically distinct. The TIA is an accessory vessel, typically originating from the brachiocephalic trunk or the aortic arch and ascending along the anterior surface of the trachea to reach the isthmus [10,11]. Conversely, the vessel described in this case exemplifies an aberrant origin of the ITA. Morphologically, it conforms to the typical distribution pattern of an ITA, entering the posterolateral aspect of the lobe, but originates directly from the CCA rather than from the thyrocervical trunk. Moreover, the TIA generally supplements the standard arterial complement [10,11], whereas in our case, it presents a compensatory morphology in which the aberrant right ITA (RITA) supplies the entire gland in the absence of the left ITA.

The variations observed here are clinically critical because the ITA serves as a primary surgical landmark for identifying the recurrent laryngeal nerve (RLN) during thyroidectomy and other anterior neck procedures [12]. The altered vascular supply in this case may increase the risk of intraoperative hemorrhage. Reviews indicate that undetected anatomical variants, particularly those with aberrant trajectories, can lead to significant bleeding and even severe complications if not identified preoperatively [3,4]. Because the RITA in this cadaver provides compensatory supply to the left lobe via an extensive isthmic network, inadvertent ligation or injury to this single vessel could compromise the blood supply of the entire gland. Furthermore, an ITA with an aberrant course over the trachea poses specific risks during procedures such as tracheostomy or laryngeal transplantation, where the vessel may be unexpectedly encountered and injured [4,7]. The relationship between the ITA and the RLN is intrinsically variable and is a major determinant of iatrogenic injury rates, which typically range from 2% to 5% even in patients without nerve variations [12]. In the present case, the aberrant origin from the CCA likely alters the standard neurovascular relationship, making the ITA a potentially unreliable landmark for nerve localization. While the en bloc removal precluded direct visualization of the RLN in this specific specimen, the literature suggests that such aberrant origins significantly alter the standard neurovascular topography.

## Conclusions

To conclude, we identified the coexistence of a rare aberrant origin and the absence of ITA in the same cadaver. Identification of even scarce arterial variants is possible preoperatively with computed tomography angiography. Surgeons should carefully review multiplanar and three-dimensional reconstructions to document the anatomy of the area.
